# Ceramide synthase-4 orchestrates the cell proliferation and tumor growth of liver cancer *in vitro* and *in vivo* through the nuclear factor-κB signaling pathway

**DOI:** 10.3892/ol.2021.12551

**Published:** 2021-02-15

**Authors:** Jinwu Chen, Xiaojie Li, Dengjiao Ma, Tao Liu, Pingping Tian, Chuanfang Wu

Oncol Lett 14: 1477-1483, 2017; DOI: 10.3892/ol.2017.6365

Subsequently to the publication of the above article, an interested reader drew to the authors’ attention that, on p. 1480, the data shown in Fig. 2A appeared to be identical with that shown in Fig. 2B.

After having re-examined their data, the authors realized that Fig. 2 had been assembled incorrectly, and the data that were intended to have shown the experiments for Fig. 2B (with Huh7 cells infected by recombinant lentivirus) had not been featured with the published figure. The revised version of Fig. 2, showing the corrected data for Fig. 2B, is shown below. Note that the regrettable but inadvertent error made in compiling this figure affected neither the results nor the conclusions reported in this paper, and all the authors agree to this Corrigendum. The authors thank the Editor of *Oncology Letters* for presenting them with the opportunity to publish this Corrigendum, and apologize to the Editor and to the readership of the Journal for any inconvenience caused.

## Figures and Tables

**Figure 2.**
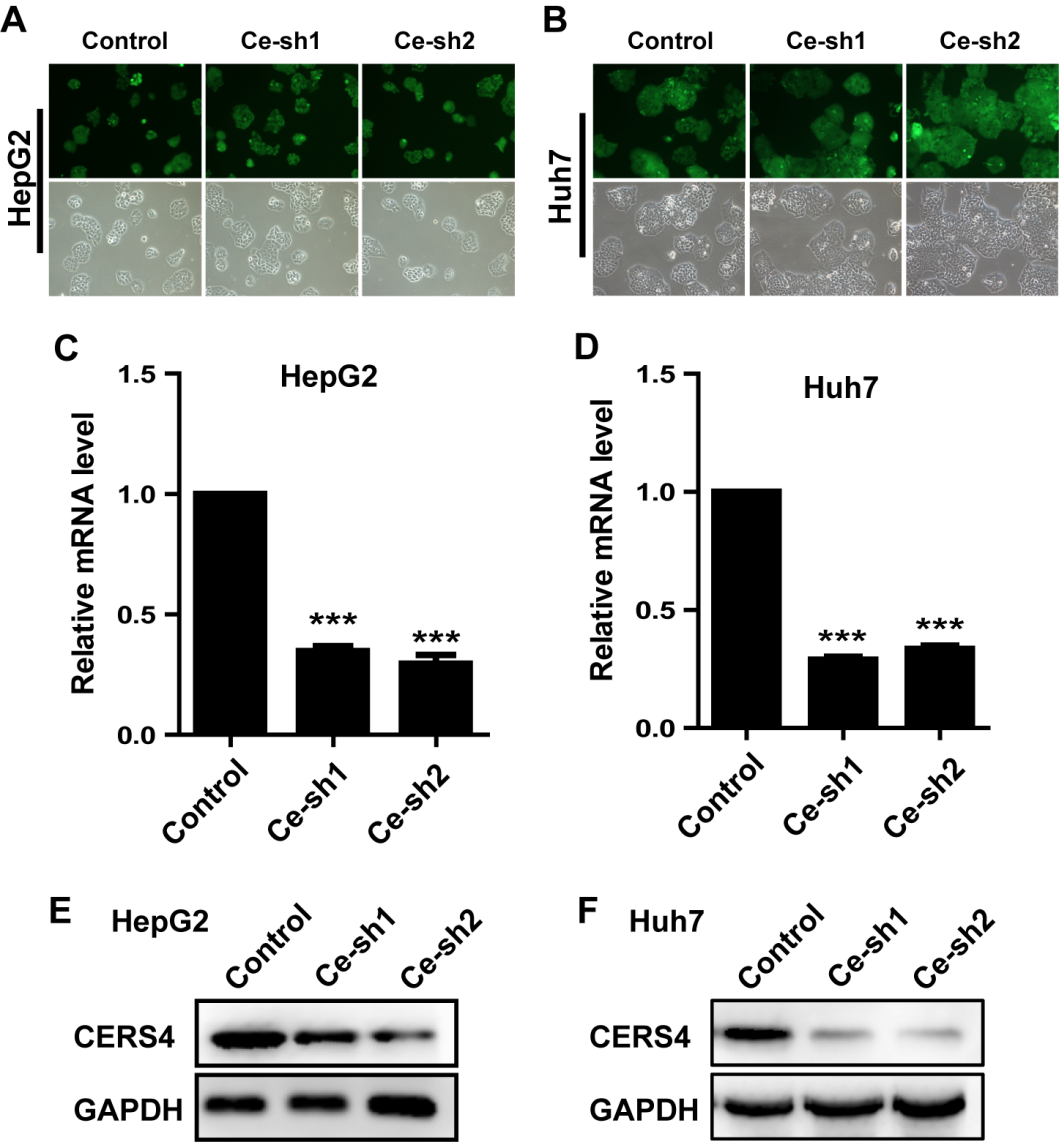
Silencing of CERS4 expression by lentivirus-mediated RNAi. Representative images of (A) HepG2 and (B) Huh7 cells infected by recombinant lentivirus. Reverse transcription-quantitative polymerase chain reaction assay to detect the knockdown efficiency of CERS4 in (C) HepG2 and (D) Huh7 cells (n=3). The knockdown efficiency of CERS4 determined by western blot analysis in (E) HepG2 and (F) Huh7 cells. The values are presented as the mean ± standard error of the mean. ***P<0.001. CERS4, ceramide synthase-4.

